# Suppression of cuelure attraction in male Queensland fruit flies provided raspberry ketone supplements as immature adults

**DOI:** 10.1371/journal.pone.0184086

**Published:** 2017-08-31

**Authors:** Humayra Akter, Saleh Adnan, Renata Morelli, Polychronis Rempoulakis, Phillip W. Taylor

**Affiliations:** 1 Department of Biological Sciences, Macquarie University, Sydney, New South Wales, Australia; 2 CAPES Foundation, Ministry of Education of Brazil, Brasilia, ‎Federal District, Brazil; CINVESTAV-IPN, MEXICO

## Abstract

Tephritid fruit flies are amongst the most damaging insect pests of horticulture globally. Some of the key fruit fly species are managed using the sterile insect technique (SIT), whereby millions of sterile males are released to suppress reproduction of pest populations. Male annihilation technique (MAT), whereby sex specific lures are used to attract and kill males, is often used to reduce wild male numbers before SIT programs commence, providing released sterile males an increased numerical advantage. Overall program efficacy might be improved if MAT could be deployed simultaneously with SIT, continuously depleting fertile males from pest populations and replacing them with sterile males. However, such ‘male replacement’ requires a means of suppressing attraction of released sterile males to lures used in MAT. Previous studies have found that exposure of some fruit flies to lure compounds as mature adults can suppress subsequent response to those lures, raising the possibility of pre-release treatments. However, this approach requires holding flies until after maturation for treatment and then release. The present study takes a novel approach of exposing immature adult male Queensland fruit flies (*Bactrocera tryoni*, or ‘Qfly’) to raspberry ketone (RK) mixed in food, forcing these flies to ingest RK at ages far younger than they would naturally. After feeding on RK-supplemented food for two days after emergence, male Qflies exhibited a reduction in attraction to cuelure traps that lasted more than 20 days. This approach to RK exposure is compatible with current practises, in which Qflies are released as immature adults, and also yields advantages of accelerated reproductive development and increased mating propensity at young ages.

## Introduction

Tephritid fruit flies are significant pests of horticulture in most regions of the world, causing direct damage to crops and restricting trade. The sterile insect technique (SIT) is used for regional management of some of the most serious fruit fly pests [1,2,3]. In SIT, millions of flies are reared in factories, sterilised, and then released into the field. Sterile males mate with females of pest populations, curtailing their reproduction such that over generations the pest population is reduced [4,5].

For species that respond to strong male lures, such as methyl eugenol or cuelure [6,7,8], the male annihilation technique (MAT) is often used before the deployment of SIT [9,10]. In MAT, devices containing a male lure and an insecticide are distributed in the field to attract and kill males. MAT reduces the number of males in the pest population, skewing the sex ratio, and this increases the effective overflooding ratio when sterile males are released. A high overflooding ratio is very desirable in SIT releases, especially when released males are inferior to wild males in mating competitiveness or ecological performance [11,12].

Because released sterile males are also attracted to male lures, MAT and SIT have traditionally been used sequentially, with MAT initially reducing the abundance of wild males before commencement of SIT. This approach creates many logistical issues as the MAT devices are long lasting and are distributed through the treated region but must be retrieved prior the SIT releases. Large advantages in overall control levels and efficiency might be achieved if MAT and SIT could be used simultaneously through the release of male flies with suppressed response to the lures used in MAT [13]. In some species there is compelling evidence that pre-prelease treatment with male lures can have a lasting effect of reduced responsiveness to those lures and might provide a basis for development of simultaneous deployment of MAT and SIT. Chambers et al. [14] exposed mature males of Mediterranean fruit fly (*Ceratitis capitata*, or ‘medfly’) to trimedlure and mature males of Oriental fruit fly (*Bactrocera dorsalis*) to methyl eugenol and in both cases found that the lure exposure reduced responsiveness to traps containing these lures. Shelly et al. [15] exposed medfly males to ginger root oil, which contains α-copaene, and found reduced attraction to trimedlure traps. Although Shelly [16] found no evidence that feeding on natural sources of methyl eugenol by mature male oriental fruit flies reduced subsequent trap capture, an earlier study [17] found that pre-release exposure of oriental fruit flies to synthesised methyl eugenol resulted in significantly reduced responsiveness to traps containing this lure over the following five days. Similarly, Shelly and Villalobos [18] found that mature male melon flies (*Zeugodacus cucurbitae*) provided a wick containing cuelure, a synthetic and more volatile analogue of naturally occurring raspberry ketone (RK) [19,20], for two hours one day prior to release showed reduced responsiveness to cuelure traps during the two days following release. In all of these previous studies flies have been exposed to lures as mature adults, but this approach is not compatible with SIT programs in which flies are released shortly after emerging as immature adults.

In the present study, we consider the potential of pre-release RK supplements in food as a means of reducing response of male Queensland fruit fly *Bactrocera tryoni* (Qfly) to cuelure, the attractant used in MAT for this species, as a step toward enabling simultaneous deployment of MAT and SIT for this species. We here adopt a recently developed approach for exposing male flies to RK [21]. Rather than waiting for flies to respond naturally to RK once mature, this approach entails mixing doses of RK in the food provided to immature flies, enabling exposure to RK over a time frame that is compatible with pre-release holding periods of SIT programs that release these flies as immature adults [22,23].

Qfly is Australia’s most damaging fruit fly pest, and in some non-endemic regions outbreaks have been managed by the sequential combination of MAT followed by SIT [22,24,25]. With increased restrictions on the use of insecticides, SIT and MAT are set to become more routine management tools [26], and significant research programs are currently under way to maximise the efficacy of these tools.

In SIT operations Qfly is usually released two to three days following emergence [22,23], and this holding period provides an opportunity for the implementation of pre-release treatments that enhance male field performance. Previous studies have highlighted the benefits of yeast hydrolysate (in addition to sugar) in the pre-release diet, as this treatment promotes reproductive development and mating performance [27,28,29,30] and results in increased numbers of sexually mature (i.e., cuelure responsive) males in the field [23]. In addition to benefits of yeast hydrolysate feeding, treatment of pupae or recently emerged adults with methoprene, a juvenile hormone analogue, has been found to accelerate development [31].

In addition to yeast hydrolysate and methoprene treatments of immature Qflies, phytochemicals, such as zingerone, and their synthetic analogues, such as cuelure, have also shown promise, but until recently these had only been considered in terms of mating performance of males that feed on these compounds as mature adults [32,33,34,35]. Immature males show little or no attraction to RK analogues, and previous studies have consequently focused on the responses of mature males. The need to wait for flies to mature before providing supplements of phytochemicals or their analogues constrains the operational viability of such supplements, and in the case of Qfly this would require holding the flies for more than a week before release [36,37]. However, RK supplements mixed in the diet of immature Qfly for just two days following emergence, approximating the usual pre-release holding period, have been found to substantially increase the mating propensity of young males [21].

In the present study we consider whether the RK treatment of immature males [21] reduces subsequent responses to cuelure, the standard lure used in MAT for this species. If the promising effects reported previously for mature male medfly, oriental fruit fly and melon fly [14,17] are also evident when RK is provided to immature Qflies, this would be an important step toward enabling simultaneous deployment of MAT and SIT without the need to significantly modify the pre-release holding period.

## Materials and methods

### Source and maintenance of flies

Qfly pupae were supplied by the New South Wales Department of Primary Industries Fruit Fly Production Facility at the Elizabeth Macarthur Agricultural Institute at Camden, New South Wales, Australia (for production details, see [38]). Pupae were kept under controlled temperature (25 ± 1°C) and relative humidity (65 ± 5%) on a 13:11 h light: dark cycle in which the first and last 30 min of the light phase were simulated dawn and dusk in which light level gradually ramped up to full output and down to darkness, respectively.

### Small field cage experiment

Approximately 2000 pupae (estimated by weight) were placed in 47.5×47.5×47.5 cm fine mesh cages (Bugdorm 44545, Megaview, Taichung, Taiwan) for adult emergence with only water provided for sustenance. Because only a few flies usually emerge on the first day, the first day of emergence was discarded, and only adults that emerged over the second day were used in the experiment.

After fly emergence, the cages were provided with water and diet (1:3 yeast hydrolysate:sugar) containing high dose (5% RK) or low dose (1.25% RK) of raspberry ketone (4-(4-Hydroxyphenyl)-2-butanone, ≥98%, Sigma–Aldrich^®^, St. Louis, MO, USA) or no RK (control) for 48 hours. RK was ground using a blender before being mixed in the diet. To differentiate among treatments, both the diet and water were mixed with food colour (blue, red or yellow, Queen Fine Foods, Australia) that was clearly visible in the gut of the flies for 2–3 days. Three millilitres of liquid dye was added to 30 g of diet so that the powdered diet became a paste that was spread over a filter paper and dried for 48 hours before being provided to the flies. Two millilitres of dye was added to 50 mL of water in a 70 mL plastic container. A sponge extending through the lid of the container carried the dyed water to the flies in each cage. The dye colour was rotated among treatments between replicates. Cages containing food and water were placed in sheltered outdoor conditions with ca. 5 m between cages of each treatment group where the flies were kept until being used in the small field cage experiments.

After 48 hours of feeding, the diet was removed from the cages. Males were removed from mixed-sex cages when 3 days old, to ensure they were virgin when used in the experiments [36,37], and placed in clean 47.5×47.55×47.5 cm fine mesh cages (Megaview Bugdorm 44545) at 700–1,000 male flies per cage. These cages were supplied with sugar and water containing the same dye that each cage had received previously.

Four small field cages (3 m diameter, 2.2 m high) were used for these experiments. An artificial tree (Ikea Fejka, ca. 0.5 m diameter and ca.1.5 m high) was placed in the middle of each field cage. Male Qflies were tested for attraction to cuelure at 5, 7, 9, 11, 13, 15, 20 and 25 days after emergence. Fifty male Qflies from each treatment group were released into each field cage 30 minutes before sunrise (i.e., 150 flies per cage on each test day). Thirty minutes later, one Lynfield trap baited with 200 μL of cuelure on a cotton wick was suspended from the ceiling of each cage. Instead of using insecticide, each trap contained a white plastic sheet that was covered with brush-on Tangle-Trap^®^ sticky coating (Tanglefoot Acquisitions, Grand Rapids, MI, USA) to capture the attracted flies. Traps were collected after sunset. Captured flies were sorted by treatment and counted. This experiment was repeated 10 times, using flies from three production batches obtained at least four weeks apart.

### Large field cage experiment

In this experiment, flies were released in four large field cages (8 m wide and 24 m long metal frame covered with white mesh, 5 m at the highest point) that contained 6 lemon trees each. To simulate an SIT release under those contained conditions, rather than releasing and testing flies at specific ages as in the small field cage experiment, in this experiment flies were released immediately after the 48-hour treatment period was complete and were then sampled over twenty days. To sustain the population of the flies during the experiment, four 1 L plastic containers with 15% sugar solution were suspended from the ceiling of each cage.

Rather than using food dyes, which do not persist for more than a few days, in this experiment the pupae were coloured with fluorescent dyes (Strong Magenta 21, Lunar Yellow 27, Stellar Green 8) (Swada, Stalybridge, UK) at a rate of 2 g of dye per litre of pupae to identify the different treatment groups. The dye was rotated among treatments between replicates. As in the previous experiment, after emergence, the cages were provided with water and diet (1:3 yeast hydrolysate:sugar) containing a high dose (5%) or low dose (1.25%) of RK or no RK (control). During the simulated pre-release holding period, the RK treated flies were kept in a different room from the control flies to avoid exposure to RK odour from the treated food, from treated flies, or from faeces and other residues from the flies [32]. After 48 hours of feeding the treated food was removed and the flies were released in large field cages. For this experiment, both males and females were released in the cages.

The experiment was repeated eight times, with four large field cages each being used twice. Four Lynfield traps were suspended in each large field cage, two containing cuelure as an attractant (‘cuelure traps’) plus malathion as killing agent and two containing malathion only without attractant (‘control traps’). To remove effects of incidental captures unrelated to cuelure attraction, the number of flies captured in the control traps was subtracted from the number captured in the cuelure traps within each field cage on each day of trap clearance.

### Statistical analysis

The number of flies from each treatment captured in traps was analysed by a mixed model ANOVA. RK treatment was treated as nominal, and Age was treated as ordinal. Batch number and Cage number were included as random effects. Interactions between RK Treatment and Age were considered, and significant interactions were explored using contrasts within the full model, using t-tests. Non-significant interaction terms were excluded from final models. All statistical tests were carried out using JMP version 10.0.0 (SAS Institute, Cary, NC, USA).

## Results

### Small field cage experiment

**Fig 1:**
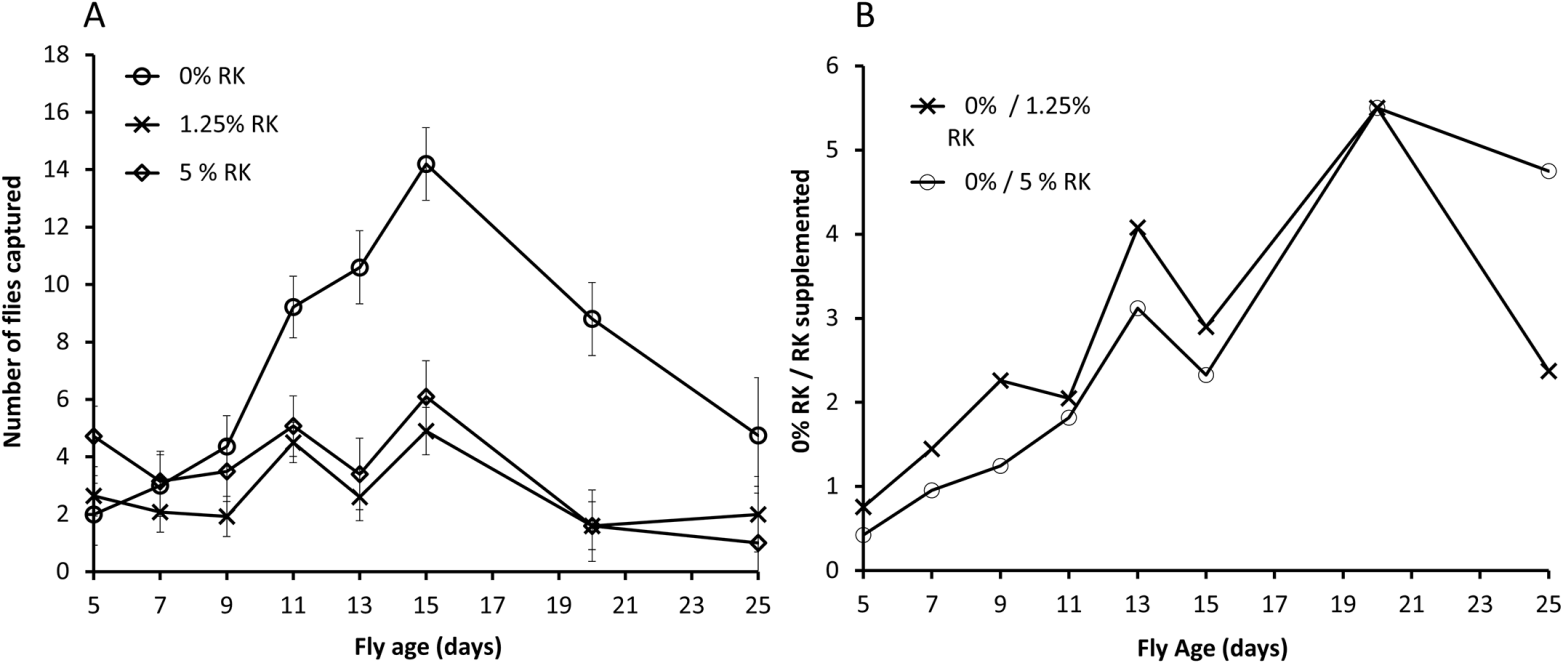
Mean number (± S.E.) of male Qflies captured in cuelure traps in small field cage trials (A) and relative number of control vs. RK treated male Qflies captured in cuelure traps in small field cage trials (B).

The effect of RK supplementation on number of flies captured in cuelure traps (square root transformed) varied across the days of testing (RK Treatment *F*_2,241.8_ = 2.510, *P* = 0.083, Age *F*_7,242.2_ = 13.874, *P*<0.001, RK Treatment x Age *F*_14,241.8_ = 5.544, *P*<0.001) (Fig 1A). Contrasts across treatments within each tested age found no evidence of differences in number of RK-supplemented and control flies captured at 5, 7, 9 and 25 days of age, the youngest and oldest ages tested, but at 11, 13, 15 and 20 days of age, the period of highest response as flies mature, significantly fewer RK-supplemented flies were captured compared with the controls. At each of these ages at which significant treatment effects were detected the contrasts between RK-supplemented treatment groups and controls were significant for both RK doses, and there were no significant differences between the two doses. At the peak of differences in daily captures when flies were twenty days of age, approximately five and a half times as many control flies were captured compared with those that received RK (Fig 1B).

### Large field cage experiment

**Fig 2:**
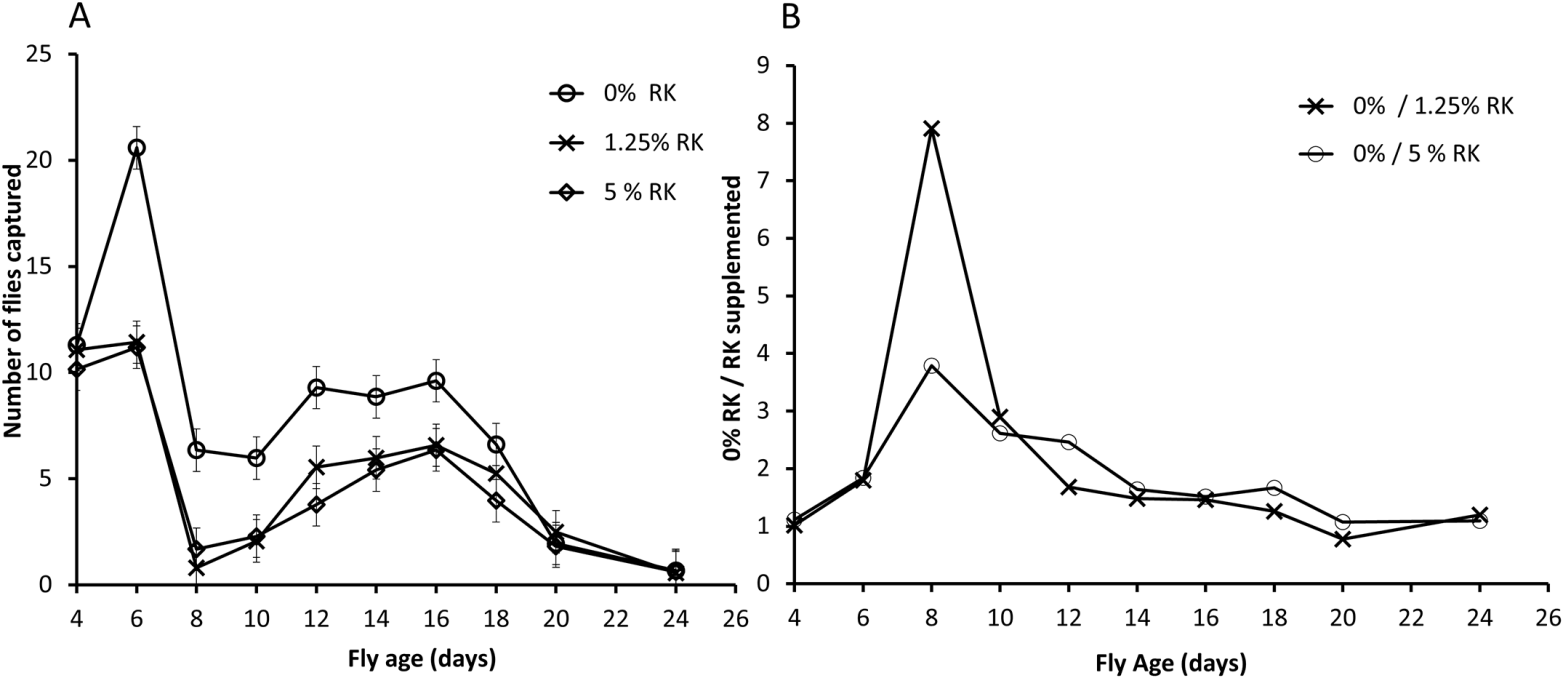
Mean number (± S.E.) of male Qflies captured in cuelure traps minus the number captured in control traps in large field cage trials (A) and relative number of control vs. RK treated male Qflies captured in cuelure traps in large field cage trials (B).

For males there was significant variation across the tested days in the corrected number of floes captured in cuelure traps (N cuelure traps—N control traps) (*F*_9,224_ = 12.409, *P*<0.001) and significant variation amongst RK treatment groups (*F*_2,224_ = 7.273, *P*<0.001), with significantly more control flies being captured than either of the RK treated groups and no difference between the RK treatments (Fig 2A). There was no evidence of variation across the tested days in the effect of RK treatment (Age*Treatment interaction *F*_18,206_ = 0.489, *P* = 0.961). At the peak of differences in daily captures when flies were eight days of age, approximately fourfold more control flies were captured compared with those that received the high RK dose and eight-fold more control flies were captured compared with those that received the low RK dose (Fig 2B).

Studies of lure attraction that follow a cohort in the field or in simulated field conditions in large outdoor cages are vulnerable to the possibility that rather than reflecting differences in attraction to a lure, the differences in number of flies from each treatment group captured reflect differences in survivorship To eliminate the possibility that our results reflect differential survivorship of RK-supplemented and control flies, we analysed the number of flies from each group captured in the control traps through the experiment as this value reflects abundance of flies in each cage independent of lure attraction. As expected, the number of flies captured in control traps declined through the experiment as abundance is reduced for all groups (*F*_9,224_ = 29.828, *P*<0.001) but there was no significant variation amongst treatment groups in the number of flies captured in control traps (*F*_2,224_ = 0.358 *P* = 0.700). There was also no evidence of differences amongst treatment groups in the rate at which number of flies captured in control traps declined through the experiment (Age*Treatment interaction *F*_18,206_ = 0.514, *P* = 0.950).

For females there was significant variation across the tested days in the number captured in cuelure traps as numbers declined through the experiment (*F*_9,224_ = 2.815, *P* = 0.004) but there was no significant variation amongst RK treatment groups (*F*_2,224_ = 0.861, *P* = 0.424). Considering only those flies captured in the control traps as a measure of survivorship of flies from the different treatment groups, there was a significant reduction in number of flies captured each day as the experiment progressed (*F*_9,224_ = 28.461, *P*<0.001) but there was no significant variation amongst RK treatment groups in the number of flies captured (*F*_2,224_ = 0.369, *P* = 0.692) and no evidence of differences amongst treatment groups in the rate at which number of flies captured in control traps declined through the experiment (Age*Treatment interaction *F*_18,206_ = 0.759, *P* = 0.746).

## Discussion

In fruit fly SIT programs, MAT is commonly used to reduce wild male numbers before the release of sterile flies [10,39,40]. However significant improvements in overall program efficacy could theoretically be achieved if it was possible to deploy MAT and SIT simultaneously, with MAT continuously depleting wild males from the field while SIT continuously replaces them with sterile males [13]. Simultaneous deployment of MAT and SIT would enable the immediate use of SIT to combat outbreaks rather than waiting until completing an MAT program before commencing the release of sterile flies and would also negate the need to retrieve MAT devices from the field before commencing SIT. Male Qflies released in SIT programs are attracted to cuelure [23], the lure used for MAT against this species [41]. For simultaneous deployment of MAT and SIT to be viable, there first needs to be an effective means of suppressing the attraction of released flies to cuelure-based MAT devices that can be implemented within operational holding and release practises. The present study identifies a potential means of achieving this.

Incorporation of RK in the diet of immature male Qfly for two days following emergence induced a persistent reduction in the number of flies captured in cuelure traps. Previous studies of oriental fruit fly, medfly and melon fly have also reported persistent reduction in responses to lures used in monitoring traps and MAT following previous exposure [14,15,17,18]. However, each of these studies entailed exposure of mature adult flies. This approach would require holding Qflies for a week or longer after emerging [37,42], which would be a significant departure from the current practises under which flies are typically released two to three days following emergence [23]. High mortality and sub-lethal stress from crowded conditions and the costs of maintaining the flies for such long periods in the rearing centres would present serious challenges to pre-release RK treatment of mature adult Qflies [25]. The present study demonstrates that effects paralleling those of previous studies of mature adults can be achieved by including RK in the diet of recently emerged immature adult male Qflies.

RK and closely related compounds have diverse effects on the behaviour and physiology when ingested by mature male Qflies. For example, ingested cuelure is incorporated in pheromone as RK [32,34], and male Qflies that ingest these compounds gain significant advantages in mating performance over the following two days. Akter et al. [21] was the first to find that RK can have significant effects even when incorporated into the diet of immature male Qflies. Male Qflies that ingest RK as immature adults exhibit greatly elevated mating propensity up to 10 days of age [21], and this tendency corresponds with significant acceleration in the development of reproductive organs over this period [43]. Usually, attraction to cuelure is associated with mature Qflies [30] and on this basis it might be expected that the accelerated development of RK-treated males would result in increased capture rates in cuelure traps at young ages. In contrast, despite being mature at younger ages, RK-fed flies exhibited significant suppression of capture rates. Amongst the youngest ages of flies tested in small field cages, the difference between capture rates of RK treated and untreated flies was not significant, with differences only becoming evident as flies matured. Rather than indicating failure of RK treatment, this reflects the low responses of flies from the control group at these ages due to them being immature. As the control flies matured their attraction to cuelure traps increased sharply, but this tendency was suppressed in the RK treated flies. There were some differences between the small cage experiment and the large cage experiment in the timing of peak response to lures, with maximum response being at around 15 days of age in the small cage experiment and six days of age in the large cage experiment. This likely reflects differences between these experimental settings in environmental conditions and survivorship. In the small cage experiment, the flies were held in a sheltered location away from direct sunlight, whereas in the large cage experiment the flies were exposed to direct sunlight, and temperature was also elevated by a greenhouse effect in the cages. Temperature-dependent differences in development rate of the flies in these two experiments are most likely a major factor driving differences in peak lure response. In the large cage experiment it is possible that high mortality rates were also an important determinant of the identified age of peak lure response, as a peak in lure response at older ages would be increasingly difficult to detect as abundance declines. It is possible that that maximum individual responsiveness to lures in the large cage experiment occurred at an age beyond the age at which the maximum number of flies was captured but that this was not evident because of a reduced number of flies available to respond. That is, the number of flies captured in the large cage experiment reflects both levels of individual responsiveness and abundance. In the small cage experiment any effect of abundance effect was removed because a standardised number of flies were tested in each trial, thereby focusing more closely on individual responsiveness.

Pre-release RK treatment shows promise as a means of suppressing attraction to cuelure traps, thereby potentially enabling the simultaneous deployment of MAT and SIT. This is a relatively simple approach, and while the effects found so far are promising they could likely be improved by further refinement of the RK-exposure protocol. In addition to pre-release RK supplements, there are alternative approaches that might be considered to suppress response of released Qflies to cuelure-based MAT devices. Selective breeding of non-responsive flies is one option, and there is some support for this possibility already in oriental fruit fly studies. Shelly [44] selected oriental fruit fly males for non-response to methyl eugenol and was able to create two lines that maintained low response to this lure over many generations. Similarly, Ito and Iwahashi [45] succeeded in selecting for lure non-responsiveness in oriental fruit fly after just two generations. Both studies concluded that while lure non-responsiveness is a quite rare trait it is amenable to selection in oriental fruit fly colonies. In the studies of Shelly [44] and Ito and Iwahashi [45] the evolution of lure non-responsiveness was considered as a risk to MAT programs. On the other hand, establishment of mass-reared populations with suppressed response to lures used in MAT would be extremely beneficial for the prospective simultaneous deployment of MAT and SIT. To date there has been no investigation of within or between population variation in male Qfly response to cuelure, and such studies are now warranted to explore this possibility. Another alternative approach might involve treatment with RNA interference (RNAi) incorporated either in larval or adult diet as a pre-release supplement. This method could be used to deactivate genes regulating the production of odorant-binding proteins responsible for the detection of cuelure and related compounds [46]. While these alternative approaches might be more effective approaches for supressing cuelure response, they are not as easily implemented and do not include the additional benefits of accelerated sexual development that has been found when feeding RK to immature males. Even if an alternative and more effective approach to suppressing cuelure response of released flies was found, there might still be a role for pre-release RK supplements to yield addition benefits in terms of accelerated development of increased mating performance [21,43].

While there would be significant advantages to SIT efficacy if MAT could be deployed simultaneously, there may also be disadvantages that should be considered in an overall program setting. Usually the abundance of mature released flies in the field is monitored using cuelure traps, and these data can be used as an indirect measure of sterile fly field performance [47]. Suppression of responses to cuelure traps would impede monitoring of released sterile flies. To counter this limitation, it might be possible to develop a calibration for the abundance of RK treated flies that would estimate an equivalency in terms of current practises. Also, under some conditions alternative monitoring systems, such as yellow sticky traps [48], could be used, although this would result in significantly increased labour costs. Under many conditions, however, the constraint on ability to monitor released flies may be a minor consideration next to the substantial potential increases in overall program efficacy [13].

## References

[1] Orozco-Dávila D, Hernández R, Meza S, Domínguez J. Sexual competitiveness and compatibility between mass-reared sterile flies and wild populations of *Anastrepha ludens* (Diptera: Tephritidae) from different regions in Mexico. Fla Entomol. 2007; 90: 19–26.

[2] Hendrichs J, Robinson A, Cayol J, Enkerlin W. Medfly area wide sterile insect technique programmes for prevention, suppression or eradication: the importance of mating behavior studies. Fla Entomol. 2002; 85: 1–13.

[3] Enkerlin W, Gutiérrez-Ruelas JM, Cortes AV, Roldan EC, Midgarden D, Lira E et al Area freedom in Mexico from Mediterranean fruit fly (Diptera: Tephritidae): A review of over 30 years of a successful containment program using an integrated area-wide SIT approach. Fla Entomol. 2015; 98: 665–681.

[4] Knipling E. Possibilities of insect control or eradication through the use of sexually sterile males. J Econ Entomol. 1955; 48: 459–462.

[5] Krafsur ES. Sterile insect technique for suppressing and eradicating insect population: 55 years and counting. J Agric Entomol. 1998; 15: 303–317.

[6] Beroza M, Alexander B, Steiner L, Mitchell WC, Miyashita DH. New synthetic lures for the male melon fly. Science. 1960; 131: 1044–1045. doi: 10.1126/science.131.3406.1044 17808098 10.1126/science.131.3406.1044

[7] Bateman M. The ecology of fruit flies. Ann Rev Entomol. 1972; 17: 493–518.

[8] Cunningham RT, Suda DY. Male annihilation through mass-trapping of male flies with methyl eugenol to reduce infestation of oriental fruit fly (Diptera: Tephritidae) larvae in papaya. J Econ Entomol. 1986; 79: 1580–1582.

[9] Bateman M, Friend A, Hampshire F. Population suppression in the Queensland fruit fly, *Dacus* (*Strumeta*) *tryoni*. I. The effects of male depletion in a semi-isolated population. Crop and Pasture Sci. 1966; 17: 687–697.

[10] Barclay HJ, Hendrichs J. Models for assessing the male annihilation of *Bactrocera* spp. with methyl eugenol baits. Ann Entomol Soc Am. 2014; 107: 81–96.

[11] Vreysen MJB, Barclay HJ, Hendrichs J. Modeling of preferential mating in areawide control programs that integrate the release of strains of sterile males only or both sexes. Ann Entomol Soc Am. 2006; 99: 607–616.

[12] Rempoulakis P, Taret G, Haq I, Wornayporn V, Ahmad S, Tomas U, et al Evaluation of quality production parameters and mating behavior of novel genetic sexing strains of the Mediterranean fruit fly *Ceratitis capitata* (Wiedemann) (Diptera: Tephritidae). PLoS ONE. 2016; 11(6): e0157679 doi: 10.1371/journal.pone.0157679 27336737 10.1371/journal.pone.0157679 PMC4918918

[13] Barclay HJ. McInnis D, Hendrichs J. Modeling the area-wide integration of male annihilation and the simultaneous release of methyl eugenol-exposed *Bactrocera* spp. sterile males. Ann Entomol Soc Am. 2014; 107: 97–112.

[14] Chambers D, Ohinata K, Fujimoto M, Kashiwai S. Treating tephritids with attractants to enhance their effectiveness in sterile-release programs. J Econ Entomol. 1972; 65: 279–282.

[15] Shelly TE, Edu J, Pahio E. Exposure to ginger root oil decreases capture of male Mediterranean fruit flies (Diptera: Tephritidae) in trimedlure-baited traps. Proc Hawaiian Entomol Soc. 2007; 39: 27–32.

[16] Shelly TE. Trapping male Oriental fruit flies (Diptera: Tephritidae): Does feeding on a natural source of methyl eugenol reduce capture probability? Fla Entomol. 2000; 83: 109–111.

[17] Shelly TE. Consumption of methyl eugenol by male *Bactrocera dorsalis* (Diptera: Tephritidae): low incidence of repeat feeding. Fla Entomol. 1994; 77: 201–208.

[18] Shelly TE, Villalobos EM. Cue lure and the mating behavior of male melon flies (Diptera: Tephritidae). Fla Entomol. 1995; 78: 473–482.

[19] Park SJ, Morelli R, Hanssen BL, Jamie JF, Jamie IM, Siderhurst MS et al Raspberry ketone analogs: Vapour pressure measurements and attractiveness to Queensland fruit fly, *Bactrocera tryoni* (Froggatt) (Diptera: Tephritidae). PLoS One. 2016a; 11(5): e0155827 27196605 10.1371/journal.pone.0155827 PMC4873134

[20] Park SJ, Siderhurst MS, Jamie IM & Taylor PW. Hydrolysis of Queensland fruit fly, *Bactrocera tryoni* (Froggatt), attractants: kinetics and implications for Biological activity. Aust J Chem. 2016b; 69: 1162–1166.

[21] Akter H, Mendez V, Morelli R, Perez J, Taylor PW. Raspberry ketone supplement promotes early sexual maturation in male Queensland fruit fly, *Bactrocera tryoni* (Diptera: Tephritidae). In press. Pest Manag Sci. 10.1002/ps.4538 28139095

[22] Dominiak BC, Westcott AE, Barchia IM. Release of sterile Queensland fruit fly, *Bactrocera tryoni* (Froggatt) (Diptera: Tephritidae), at Sydney, Australia. Aust J Exp Agric. 2003; 43: 519–528.

[23] Reynolds OL, Orchard BA, Collins SR, Taylor PW. Yeast hydrolysate supplementation increases field abundance and persistence of sexually mature sterile Queensland fruit fly, *Bactrocera tryoni* (Froggatt). Bull Entomol Res. 2014; 104: 251–261. doi: 10.1017/S0007485313000758 24456807 10.1017/S0007485313000758

[24] Monro J, Osborn A. The use of sterile males to control populations of Queensland fruit fly *Dacus tryoni* (Frogg) (Diptera: Tephritidae) I. Methods of mass rearing, transporting, irradating and releasing sterile flies. Aust J Zool. 1967; 15: 461–473.

[25] Meats AW, Clift AD, Dominiak BC. Trials on variants of the Sterile Insect Technique (SIT) for suppression of populations of the Queensland fruit fly in small towns neighbouring a quarantine (exclusion) zone. Aust J Exp Ag. 2003; 43: 389–395.

[26] Dominiak BC, Ekman JH. The rise and demise of control options for fruit fly in Australia. Crop Prot., 2013; 51: 57–67.

[27] Pérez-Staples D, Harmer AMT, Collins SR, Taylor PW. Potential for pre-release diet supplements to increase the sexual performance and longevity of male Queensland fruit flies. Agric Forest Entomol. 2008; 10: 255–262.

[28] Pérez-Staples D, Weldon CW, Smallridge C, Taylor PW. Pre-release feeding on yeast hydrolysate enhances sexual competitiveness of sterile male Queensland fruit flies in field cages. Ent Exp Appl. 2009; 131:159–166.

[29] Pérez-Staples D, Weldon CW, Taylor PW. Sex differences in developmental response to yeast hydrolysate supplements in adult Queensland fruit fly. Entomol Exp Appl. 2011; 141: 103–113.

[30] Weldon CW, Pérez-Staples D, Taylor PW. Feeding on yeast hydrolysate enhances attraction to cue-lure in Queensland fruit flies, *Bactrocera tryoni*. Entomol Exp Appl. 2008; 129: 200–209.

[31] Collins SR, Reynolds OL, Taylor PW. Combined effects of dietary yeast supplementation and methoprene treatment on sexual maturation of Queensland fruit fly. J Insect Physiol. 2014; 61: 51–57. doi: 10.1016/j.jinsphys.2014.01.002 24424344 10.1016/j.jinsphys.2014.01.002

[32] Tan KH, Nishida R. Incorporation of raspberry ketone in the rectal glands of males of the Queensland fruit fly, *Bactrocera tryoni* Froggatt (Diptera: Tephritidae). Appl Entomol Zool. 1995; 30: 494–497.

[33] Kumaran N, Balagawi S, Schutze MK, Clarke AR. Evolution of lure response in tephritid fruit flies: phytochemicals as drivers of sexual selection. Anim Behav. 2013; 85: 781–789.

[34] Kumaran N, Hayes RA, Clarke AR. Cuelure but not zingerone make the sex pheromone of male *Bactrocera tryoni* (Tephritidae: Diptera) more attractive to females. J Insect Physiol. 2014; 68: 36–43. doi: 10.1016/j.jinsphys.2014.06.015 25010549 10.1016/j.jinsphys.2014.06.015

[35] Kumaran N, Prentis PJ, Mangalam KP, Schutze MK, Clarke AR. Sexual selection in true fruit flies (Diptera: Tephritidae): transcriptome and experimental evidences for phytochemicals increasing male competitive ability. Mol Ecol. 2014; 23: 4645–4657. doi: 10.1111/mec.12880 25112896 10.1111/mec.12880

[36] Vijaysegaran S, Walter G, Drew R. Influence of adult diet on the development of the reproductive system and mating ability of Queensland fruit fly *Bactrocera tryoni* (Froggatt)(Diptera: Tephritidae). J Trop Agric Food Sci. 2002; 30: 119–136.

[37] Pérez-Staples D, Prabhu V, Taylor PW. Post-teneral protein feeding enhances sexual performance of Queensland fruit flies. Physiol Entomol. 2007; 32: 225–232.

[38] Dominiak BC, Sundaralingam S, Jiang L, Jessup A, Barchia I. Production levels and life history traits of mass reared Queensland fruit fly *Bactrocera tryoni* (Froggatt)(Diptera: Tephritidae) during 1999/2002. Plant Prot Q. 2008; 23: 131.

[39] Steiner L, Hart W, Harris E, Cunningham R, Ohinata K, Kamakahi D. Eradication of the oriental fruit fly from the Mariana Islands by the methods of male annihilation and sterile insect release. J Econ Entomol. 1970; 63: 131–135.

[40] Koyama J, Teruya T, Tanaka K. Eradication of the oriental fruit fly (Diptera: Tephritidae) from the Okinawa Islands by a male annihilation method. J Econ Entomol. 1984; 77: 468–472.

[41] Dominiak BC, Ekman J, Broughton S. Mass trapping and other management options for Mediterranean fruit fly and Queensland fruit fly in Australia. Gen Appl Entomol. 2016; 44: 1–8.

[42] Meats A, Holmes H, Kelly G. Laboratory adaptation of *Bactrocera tryoni* (Diptera: Tephritidae) decreases mating age and increases protein consumption and number of eggs produced per milligram of protein. Bull Entomol Res. 2004; 94: 517–524. 15541191 10.1079/ber2004332

[43] Akter H. Raspberry ketone as a promising pre-release supplement for the Sterile Insect Technique (SIT) of Queensland fruit fly, Bactrocera tryoni (Froggatt) (Diptera: Tephritidae). 2017. PhD Thesis, Macquarie University.

[44] Shelly TE. Selection for non-responsiveness to methyl eugenol in male oriental fruit flies (Diptera: Tephritidae). Fla Entomol. 1997; 80: 248–253.

[45] Ito Y, Iwahashi O. Ecological problems associated with an attempt to eradicate Dacus dorsalis (Tephritidae: Diptera) from the southern islands of Japan with a recommendation on the use of sterile male technique, pp. 45–53 in 1974 Proceedings of a panel on the sterile insect technique and its field applications. International Atomic Energy Agency-PL-494/5.

[46] Siciliano P, He XL, Woodcock C, Pickett JA, Field LM, Birkett MA et al Identification of pheromone components and their binding affinity to the odorant binding protein CcapOBP83a-2 of the Mediterranean fruit fly, *Ceratitis capitata*. Insect Biochem Mol Biol. 2014; 48: 51–62. doi: 10.1016/j.ibmb.2014.02.005 24607850 10.1016/j.ibmb.2014.02.005 PMC4003389

[47] Meats A. A quality assurance measure for field survival rates of released sterile flies based on recapture rates. Gen Appl Entomol. 1998; 28: 39–46.

[48] Weldon CW, Meats AW. Short-range dispersal of recently emerged males and females of *Bactrocera tryoni* (Froggatt) (Diptera: Tephritidae) monitored by sticky sphere traps baited with protein and Lynfield traps baited with cue-lure. Aust J Entomol. 2007; 46: 160–166.

