# Correction: Suppression of cuelure attraction in male Queensland fruit flies provided raspberry ketone supplements as immature adults

**DOI:** 10.1371/journal.pone.0191145

**Published:** 2018-01-09

**Authors:** Humayra Akter, Saleh Adnan, Renata Morelli, Polychronis Rempoulakis, Phillip W. Taylor

In the Funding section, the grant number from the funder Hort Frontiers Fruit Fly Fund is listed incorrectly. The correct grant number is: HG14033.
